# Effect of simulator frame rate on pilot training for high-speed fighter aircraft

**DOI:** 10.3389/fphys.2026.1763485

**Published:** 2026-03-27

**Authors:** John Jong-Jin Kim, Ramy Kirollos

**Affiliations:** Defense Research and Development Canada, Toronto Research Center, Toronto, ON, Canada

**Keywords:** fast jets, flight simulators, frame rate, training, visual displays

## Abstract

The current generation of fighter aircraft (e.g., F-35) move at supersonic speeds. Fighter pilots undergo rigorous simulation-based training to adapt and learn to operate these high-speed aircraft. On a simulator’s display of a cockpit view of a moving aircraft, a fast-moving scene or objects seen by the pilot is presented as a series of computer-generated images. Due to the computing time for each image frame to be rendered, there are gaps between each frame which may present as visual artifacts in the virtual scene. This problem becomes amplified the faster objects move on the display. The missing visual information between frames presented can be detrimental for fast-paced high-intensity tasks, especially at low frame rates. Therefore, the effect of frame rate of the flight training simulator needs to be carefully evaluated to avoid negative transfer of training and ensure pilot safety. This paper reviews the current literature related to frame rate on viewer preference, its influence on task performance, simulator sickness and training. The aim of this paper was to identify gaps in scientific knowledge on the impact of simulator frame rate and provide future research directions to investigate pilot learning and transfer of complex tasks with extremely fast-moving scenes or objects across simulated and real fighter aircraft.

## 1 Introduction

In the era of supersonic fighter aircraft such as F-35 (5th generation) and Gripen (4.5th generation), it is becoming exceedingly important to understand the human factors related to the pilot’s operation of these advanced aircraft. The human body is not accustomed to the supersonic speed (generally measured in Mach) and extreme acceleration (generally measured in g-force, i.e., G) involved with operating such aircraft. This can result in unexpected outcomes in the pilots’ health, cognition and performance due to these extreme physical and mental stressors. Pilots go through rigorous training to adapt and learn to handle such stress and to operate these aircraft. For such training to be safe and effective, the flight training organization must ensure that their training system or program is designed to avoid transferring negative learning from simulation-based training to real aircraft operations.

Simulator-based training has been an effective and efficient alternative to in-flight pilot training for decades since its rise in the 1970s (“Aviation Training Device Credit for Pilot Certification”, 2016). In the last decade, simulators using virtual reality (VR) head-mounted displays (HMD) have also been introduced and implemented into pilot training curricula. For instance, VR HMD-based training was formally implemented in the United States Department of Defense for their ab initio (introductory) pilot training (Mishler et al., 2022). Airlines in the European Union begun accrediting VR HMD-based simulators in 2024 (Loft Dynamics, 2024). The emerging interest in implementing new extended reality HMD technologies and high fidelity world-fixed displays (e.g., 2D monitors and projector-based displays) into training simulators calls for more rigorous research on the impact of frame rate, refresh rate, resolution, and motion-to-photon lag of the display systems on pilot training. This paper presents current scientific literature related to the display FR on how they might impact human task performance and training effectiveness. We aim to evaluate what is known in scientific literature, identify any gaps in knowledge, and provide directions for future research that will investigate how FR might influence pilot trainees when dealing with extremely fast-moving scenes or objects in simulators.

### 1.1 Frame rate and refresh rate

Typically, a flight simulator employs either proprietary or commercial software (e.g., Unreal Engine) to run a virtual simulation of an aircraft in a 3D environment. Then a series of images visually depicting the simulated environment are rendered and projected on the simulator display. The frame rate (FR) is the number of new graphical renderings generated each second, typically measured in frames per second (fps). Refresh rate is the number of times renderings are re-drawn on the display each second, typically measured in Hertz (Hz).

**Figure 1:**
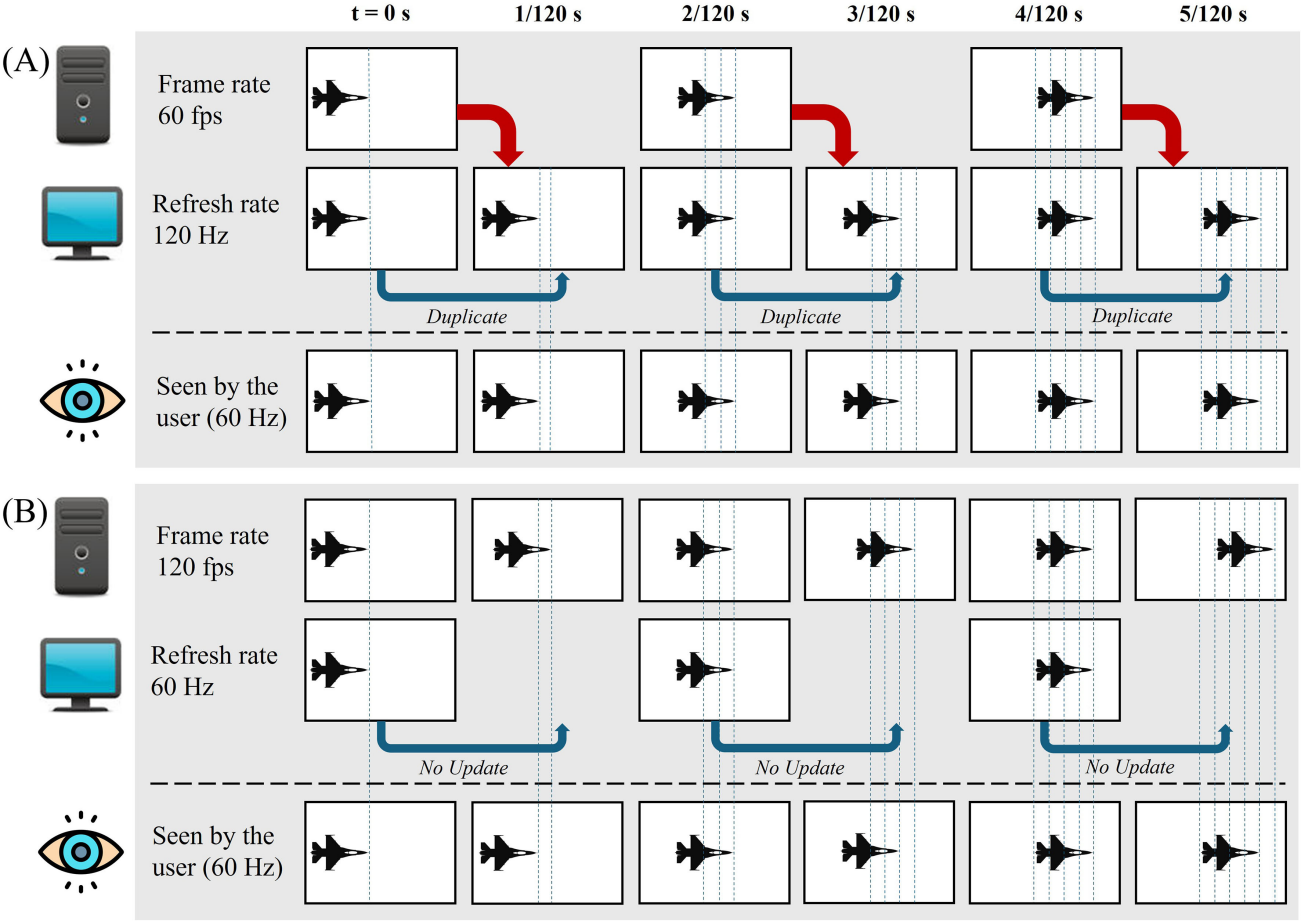
Illustration of the display seen by the user with an unequal frame rate and refresh rate. Example **(A)** demonstrates images generated at 60 fps and projected at 120 Hz. Example **(B)** demonstrates 120 fps displayed at 60 Hz. The dotted vertical lines represent the motion of the object in the images at each time frame (t) in seconds (s). These lines illustrate the comparison of the images generated, updated on the display, and seen by the user. In both examples, the changes in the images seen by the user are at 60 Hz due to either duplicated frame [i.e., projecting the same image twice, such as in **(A)**], or no frame update [i.e., the image is projected for a longer time, such as in **(B)**].

FR and refresh rate are not always the same. The FR is driven by the system’s computing power and varies depending on the complexity of the frame being rendered (e.g., number of entities and their details in the scene) whereas the refresh rate is generally fixed for the display (the updating rate of the projector or the monitor). The change of images projected on a display is governed by the slower rate of the two. Examples are given in **Figure 1**.

This review focuses on FR and assumes a simulator display with a refresh rate equal to, or higher than the FR. Therefore, we use the term ‘FR’, and use ‘Hz’ as the unit throughout this paper.

### 1.2 Simulated aircraft motion on low FR displays

Consider this: A supersonic aircraft moving at the speed of Mach 1.6 (~1,970 km/h), the maximum speed of F-35 (Lockheed Martin Corporation, 2023), simulated on a 60 Hz display (delay of ~0.017 s between frames) moves about 9 m between each frame. Depending on the tasks at hand, this gap of missing visual information can be detrimental during high-intensity missions such as air-to-air engagement, formation flying, air-to-air refueling, and low-level flight. Might the simulation of the 5th- or 6th-generation fighter aircraft tasks (i.e., scenes or objects moving at extreme speed) displayed with 60–120 Hz, typical FR of modern simulator displays, have a negative impact on the trainee performance and/or training?

## 2 Literature review

To answer this question, a literature search was conducted using the ‘snowball’ search method between Oct. 31^st^ – Nov. 14^th^, 2025. Google Scholar and Institute of Electrical and Electronics Engineers (IEEE) databases were queried for peer-reviewed journal or conference papers using the following keyword searches: ‘frame rate’ combined with ‘quality’, ‘preference’, ‘performance’ or ‘training’. From the result of each keyword search, the first 2–3 articles that were accessible were evaluated for their relevance. Review papers and meta-analysis that reviewed multiple articles were first assessed. Articles that were not directly related to the keywords, or did not involve human participants (e.g., studies using computer camera systems) were not selected for the review. After reviewing the papers, other relevant works identified from the paper were searched from their reference list. Papers were categorized into four main topics: 1) Viewer FR preference, 2) Objective task performance, 3) Transfer of training (ToT) from simulated to real-world applications, and 4) Display advancements and simulator sickness based on findings from our search terms. No studies were found specifically examining FR in flight simulation.

### 2.1 Perceived FR quality and preference

**Figure 2:**
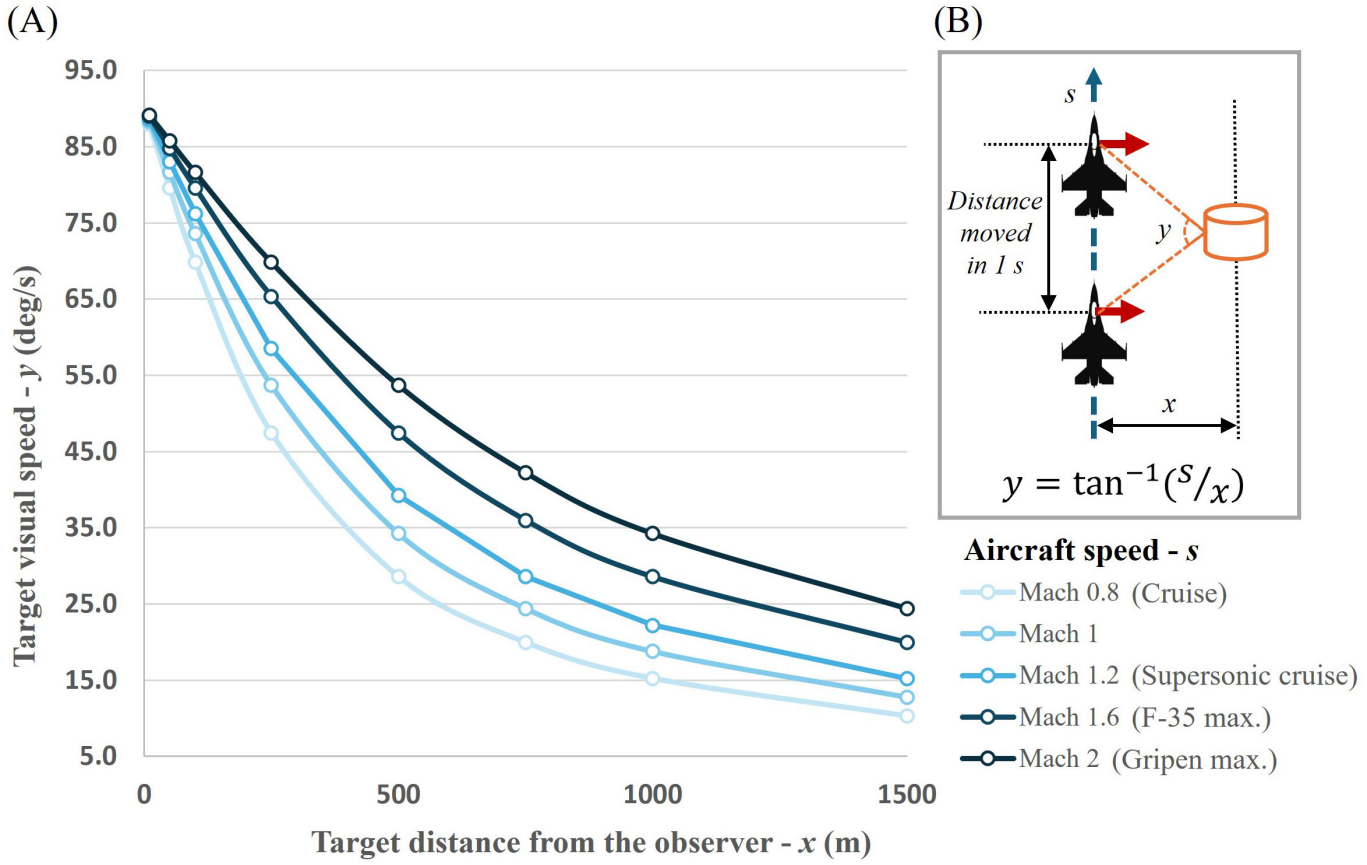
Visual angular speed of an object at varying distances seen by the pilot. In graph **(A)**, each line represents the speed of the target seen by the pilot [*y*; shown in **(B)**] moving at different aircraft speeds (*s*). The relevant speed of a target object in the observer’s visual field depends on its distance from them (*x*). Diagram **(B)** shows the formula and the parameters used to plot graph **(A)**. In this case, a pilot flying in the direction of the blue dashed arrow line is looking out to the side (in the direction of the red arrows) seeing a stationary target (orange cylindrical building).

Past studies show people watching videos prefer higher FR (48–60 Hz) over lower FR (24 Hz) videos (Wilcox et al., 2015). They also perceive video clips with higher FRs (up to 120 Hz) as higher in quality than lower FR (as low as 15 Hz) video clips (Mackin et al., 2018), likely due to fast-moving objects projected in low FR appearing blurry or choppy. People can detect motion artifacts, such as motion blur, at FRs up to 600 Hz and these artifacts become more noticeable when objects in the scene move at faster speeds (Mackin et al., 2017). Hence, when the simulations of the current generation fighter aircraft moving at supersonic speed are projected on simulator displays, it is likely that the motion artifacts from the rapidly changing scene or objects are noticeable to the users. Reports of visually induced sensation of self-motion known as *vection* are enhanced with higher FR (Weech et al., 2020). For instance, Weech et al. found greater body sway in people viewing forward optic flow on projection screen at 480 Hz than in FRs lower than 240 Hz. Changes in the visual scene during a flight, whether in a real flight or on a flight simulator’s display, depends on various parameters (e.g., aircraft speed, object distance, viewing angle, etc.). An example of the relationship between the visual speed of a target object seen by the pilot and the target distance is presented in **Figure 2**.

Mackin et al. (2017) suggested FRs needed for the motion artifact to become imperceptible to the viewers (critical frame rate; CFR) for various stimulus speeds based on their experiment. At 10 deg/s, the slowest speed in **Figure 2A**, the CFR estimated by Mackin et al. was around 100 Hz. This means people using a simulator with FR less than 100 Hz, for the particular example case (**Figure 2B**), can perceive motion artifacts of any moving objects within 1.5 km with a simulated speed faster than Mach 0.8. At the fastest speed tested at 70 deg/s, the CFR was approximately 600 Hz - much higher than FRs generally used in standard flight simulators.

FRs of at least 100 Hz were recommended for conventional entertainment systems (Mackin et al., 2017) and 120 Hz for VR HMDs (Wang et al., 2023). However, supersonic aircraft flight simulators require higher FR than those used for everyday purposes to be perceived by pilots without any visual artifacts. Studies presented here demonstrate a general preference for high FR content, but they did not assess FR effects on performance and training effectiveness.

### 2.2 The impact of FR on task performance

Chen and Thropp (2007) reviewed over 50 studies on the effect of low FR on human performance during various tasks. Most studies used FRs below 40 Hz, but six studies tested up to 60 Hz and one study up to 160 Hz. The review showed that higher FR generally increased performance in simple experimental tasks (e.g., target tracking and target recognition tasks), but the performance usually peaked at FRs below 35 Hz (in some cases as low as 5 Hz) then stabilized. Chen and Thropp’s review revealed that task performance effect of FR greatly depended on the type of task. For example, Lai and Duh (2004) found that when judging the percentage increase in length of a horizontal 3-D bar, alternating between two lengths, people were most accurate in the 10–40 Hz range and least accurate at 160 Hz. Such findings demonstrate the importance of testing user performance on tasks relevant to the purpose of the simulators (e.g., aircraft operation) to accurately evaluate the effect of FR.

Claypool and Claypool (2007) found increased scores in a first-person shooting task (moving a crosshair with a mouse to targets to shoot them) with higher FRs (30 and 60 Hz) than with lower FRs (3–15 Hz). Wang et al. (2023) found that people using VR HMD were more accurate and faster when hitting a target, by swinging a controller, or choosing a correct orientation of a target approaching them with high FRs (up to 180 Hz) than with a low FR (60 Hz). These studies verify past findings that user task performance generally benefits from higher FRs (Chen and Thropp, 2007). But these studies mostly involved simple visual tasks or tasks in a gaming environment, not in the aviation context. Hence, their findings have limited applicability for flying supersonic aircraft. To properly evaluate the effect of FR on the flight training simulators, the tasks relevant to their specific purposes must be identified and their performance tested against various FRs.

### 2.3 The impact of FR on training effectiveness

In our search, we identified a gap in the literature on the effect of FR on training effectiveness. Training effectiveness is often measured by ToT experimental research design (see AGARD, 1980; Korteling et al., 2013; Kirollos et al., 2025 for recent relevant discussions on ToT designs). ToT can either be negative (i.e., performance on the actual task is worse because of rehearsal in the training medium), positive (i.e., performance on the actual task is better because of rehearsal in the training medium) or neutral (i.e., no difference in performance because of rehearsal in training medium). Much of the fundamental research on high-fidelity simulators, known as flight training devices, agree that they generally lead to positive ToT (Callender, 2008; Judy and Gollery, 2019; Macchiarella et al., 2008; Stewart et al., 2002; Taylor et al., 1999, Taylor et al., 2003). But, no past research, to our knowledge, has investigated ToT with varying display FRs and its impacts.

Past experience with low FR devices (e.g., early-stage VR HMDs) have been shown to reduce the negative impact of low FR in task performance, accuracy and reaction time in psychophysical and gaming tasks (Chen and Thropp, 2007). This suggests that exposure to low FR can influence the user’s future performance as they may learn inappropriate compensation strategies. Wang et al. (2023) also found that when people look at fast-moving objects at 60 Hz, they adopt strategies, such as predicting or filling missing gaps, to compensate for the lack of visual detail. Adopting such strategies may demand higher mental workload for the simulator users during tasks and could produce negative ToT.

### 2.4 The impact of FR on visual display technology advances and simulator sickness

Out of the 50+ studies reviewed by Chen and Thropp (2007), four mentioned using head-tracked 3D stereoscopic world-fixed displays for their experiments, three studies used VR HMDs, and the remaining studies used regular 2D monitors. Claypool and Claypool (2007) used a 2D monitor in their task and Wang et al. (2023) used a VR HMD. Considering most flight simulators use cross-cockpit collimated displays (a high-fidelity display system used in full flight simulators), or VR HMD with a virtual cockpit in more recent simulators, there is a disconnect in the visualization modality between the training simulators and the available literature on FR. Hence, findings from past studies, mostly conducted on 2D monitors, may not match the human performance requirements for current and future flight simulator displays accurately.

FR is identified as one of the display-related factors contributing to simulator sickness (Lawson et al., 2021) where viewing a display with lower FR generally produces more severe sickness than viewing higher FR displays (Wang et al., 2023). De Winkel et al. (2022) conducted a meta-analysis on 41 simulator studies (FRs up to 500 Hz) finding that people experienced stronger simulator sickness in HMDs than 2D monitors or cross-cockpit displays. These findings may suggest that the impact of FR differs between visual display technologies. For example, the effect of lower FR may be more apparent in HMDs than on monitors, but this relationship needs to be further researched.

## 3 Discussion

In all, the results from presented research investigating the effect of FR on task performance generally favors higher FR for simulation-based flight training. But the experiments used non-aviation related tasks, and were mostly conducted on 2D monitors, not cross-cockpit displays or HMDs commonly used in flight training simulators. In addition, we found a gap in the literature on how FR affects ToT. Therefore, the current scientific knowledge fails to draw a complete picture on the effect of FR on pilot simulator training.

The extreme speeds simulated and projected on a display result in the scene or objects moving multiple meters between frames. People develop prediction strategies to deal with this missing visual information (Wang et al., 2023). But it is largely unknown how learning and adopting strategies due to limited FR on the simulator display can impact perceived mental workload and transfer (positive or more likely negative) to real-life tasks. For pilots operating supersonic aircrafts, potentially engaging in fast-paced high-intensity missions often requiring extreme accuracy and precision, any negative ToT may be critical for their safety.

### 3.1 Future research directions

Preliminary fundamental lab-based tasks comparing performance and ToT at different FRs should first be assessed to inform future research on simulated aircraft tasks. To address the effect of FR on the specific applications of flight simulator training, especially for the current 5^th^- and future 6^th^-generation fighter aircrafts, tasks relevant to each specific use case (e.g., air-to-air engagement, air-to-air refueling, visual pursuit, formation flying, landing, low-level flying or weapons engagement training) should be identified. The aircraft speeds (e.g., cruise speed or combat speed) for each task should critically be identified and simulated for experiments.

Then, these tasks should be compared in simulators with varying FR to study task performance and ToT. Using a method like Detection-Response Task, where participants respond to an audio or tactile stimuli every 3–5 seconds, or self-report questionnaires (e.g., NASA task load index; Sepehr, 1988), may be beneficial to quantify the possible mental workload added with adopting prediction strategies to compensate for the low FR. These studies should use high-fidelity cross-cockpit displays or HMDs to establish direct link to the visual display technology of the simulator. Research in these areas should also monitor simulator sickness to ensure it is mitigated to the extent possible based on FR manipulations.

### 3.2 Factors to consider as trade-offs to FR

To project images on a display at a stable FR, each frame must be generated within a sufficient frame time (FT) – the time it takes for the computer to generate a new image frame – for that given FR (e.g., FT of 0.017 s or shorter for 60 Hz). The required computing power varies from frame to frame, depending on the number of entities and the details of the scene. Chen and Thropp (2007) stated that it is important to consider both average and variance (i.e., standard deviation) of FT when considering FR. Spikes (i.e., sudden increases) in FT in a simulation can result in unstable FR (i.e., high variability) which can reduce user immersion and fidelity of the simulator.

Increasing the FR on the simulator display may also result in reduced performance in other aspects of the visual scene. Reduced display resolution and increased lag are some of the possible trade-offs in deciding what FR to employ in a simulator. It is noteworthy that high lag has shown to reduce tracking performance on a screen (Bradley and Abelson, 1995) and in VR (Ellis et al., 1999) displays. Low resolution and high lag have also shown to increase user simulator sickness (De Winkel et al., 2022). Future research should consider these adjacent factors to FR which can also influence user performance and possibly ToT.
